# Three-dimensional clinical assessment for MRONJ risk in oncologic patients following tooth extractions

**DOI:** 10.1259/dmfr.20230238

**Published:** 2023-10-24

**Authors:** Catalina Moreno Rabie, Rocharles Cavalcante Fontenele, Nicolly Oliveira Santos, Fernanda Nogueira Reis, Tim Van den Wyngaert, Reinhilde Jacobs

**Affiliations:** 1 OMFS-IMPATH Research Group, Department of Imaging and Pathology, Faculty of Medicine, University of Leuven, Leuven, Belgium; 2 Department of Oral and Maxillofacial Surgery, University Hospitals Leuven, Leuven, Belgium; 3 Department of Oral Diagnosis, Division of Oral Radiology, Piracicaba Dental School, University of Campinas, Piracicaba, São Paulo, Brazil; 4 Department of Nuclear Medicine, Antwerp University Hospital, Edegem, Belgium; 5 Faculty of Medicine and Health Sciences, University of Antwerp, Antwerp, Belgium; 6 Department of Dental Medicine, Karolinska Institutet, Stockholm, Sweden

**Keywords:** Diphosphonates, Denosumab, Tooth Extraction, Osteonecrosis, Cone-Beam Computed Tomography

## Abstract

**Objectives:**

To identify clinical and local radiographic predictors for medication-related osteonecrosis of the jaws (MRONJ) by the assessment of pre-operative CBCT images of oncologic patients treated with anti-resorptive drugs (ARDs) undergoing tooth extractions.

**Methods:**

This retrospective, longitudinal, case–control study included clinical and imaging data of 97 patients, divided into study and control group. Patients in the study group (*n* = 47; 87 tooth extractions) had received at least one dose of ARD, undergone tooth extraction(s), and had a pre-operative CBCT. An age-, gender-, and tooth extraction-matched control group (*n* = 50; 106 tooth extractions) was selected. Three calibrated, blinded, and independent examiners evaluated each tooth extraction site. Statistical analysis used χ^2^/Fisher’s exact/Mann–Whitney *U* test to contrast control and study group, ARD type used, and sites with or without MRONJ development. *p*-value ≤ 0.05 was considered significant.

**Results:**

From the study group, 15 patients (32%) and 33 sites (38%) developed MRONJ after tooth extraction. When controls were compared to study sites, the latter showed significantly more thickening of the lamina dura, widened periodontal ligament space, osteosclerosis, osteolysis, and sequestrum formation. In the study group, MRONJ risk significantly increased in patients who had multiple tooth extractions, were smokers, and had shorter drug holidays. Periosteal reaction and sequestrum formation may indicate latent MRONJ lesions. Additionally, patients given bisphosphonates showed considerably more osteosclerosis than those given denosumab.

**Conclusions:**

Periosteal reaction and sequestrum formation are suspected to be pre-clinical MRONJ lesions. Furthermore, ARD induced bony changes and radiographic variations between ARD types were seen.

## Introduction

The skeleton is one of the most common locations for metastases, with breast and prostate cancer accounting for more than 80% of their incidence. Metastatic bone disease can be accompanied by skeletal-related events (SRE), which are distressing signs and symptoms comprising pain, impaired mobility, hypercalcemia, fractures, and spinal cord compression.^1^ Fortunately, treatments are available to prevent or treat SREs, including anti-resorptive drugs (ARDs) such as bisphosphonates and monoclonal antibodies.

Among the available treatments, ARDs interfere with bone turnover by impeding osteoclastic activity through different pathways.^2,3^ For instance, nitrogen-containing bisphosphonates bind to calcium ions on the bone surface and are internalized by resorbing osteoclasts, inhibiting the protein farnesyl pyrophosphate (FPP) synthase, which is required for osteoclast function.^4,5^ On the other hand, monoclonal antibodies, like denosumab, inhibit the RANK-ligand pathway impairing osteoclast formation.^3^ Both strategies result in a reduction of bone resorption and destruction.

Patients receiving ARDs require special attention in the dental practice because of the higher risk of developing medication-related osteonecrosis of the jaws (MRONJ).^6^ MRONJ is clinically defined as exposed bone or bone that can be probed through an intraoral or extraoral fistula(e) in the maxillofacial region persisting for more than 8 weeks in patients treated with ARDs, with no history of radiation therapy or metastatic disease to the jaws.^7^ Interestingly, previous studies have reported changes in the radiographic appearance of the jawbones in these patients even before the manifestation of exposed necrotic bone. While panoramic radiographs may thus provide a good overview and early indicator of patients at risk of MRONJ, they may fall short in identifying even earlier signs due to their low sensitivity to detect minor variations.^8^ Instead, CBCT has been recommended in these patients,^9–12^ where thickening of the mandibular cortical and lamina dura,^11^ osteosclerosis,^11,13^ and osteolysis can be observed.^13^

Several risk factors for MRONJ have been acknowledged, including high cumulative doses of ARDs^14,15^ and tooth extractions.^14^ Particularly when tooth extractions take place, local predisposing factors for MRONJ have been recognized using panoramic radiographs, endorsing an increased susceptibility at sites with dental infections and osteosclerotic and osteolytic changes.^16–18^ The latter together with cortical bone erosion, sequestrum, and sinus inflammatory signs have been associated to histological evidence of osteonecrosis. Nevertheless, it remains unclear whether any of these CBCT-based features are pathognomonic for MRONJ.^12^ Therefore, the main objective of this retrospective case–control study was to identify clinical and local radiographic predictors for MRONJ using CBCT images of oncologic patients treated with ARD undergoing tooth extractions. Secondary objectives included comparing radiographic findings between patients treated with ARD *vs* those without, and between patients treated with bisphosphonates or denosumab. It was hypothesized that CBCT can provide early visualization of pre-clinical stages of MRONJ.

## Methods and material

### Study design and settings

The ethical committee of UZ/KU Leuven was consulted prior to the start of this retrospective case–control study (protocol number: S63934). All procedures were performed according to the ethical standard of the Declaration of Helsinki and the institutional review board. The STROBE guidelines were followed for reporting.^19^

### Participant selection

Clinical records of 525 patients treated with ARDs and seen at the oral and maxillofacial surgery department at University Hospitals Leuven for CBCT acquisition between 2010 and 2020 were reviewed retrospectively. Patients were included if they: (1) received ARD therapy in oncological doses, (2) had tooth extraction(s) within 1 year after CBCT acquisition, and (3) had documented clinical follow-up of the extraction socket. Patients with prior head and neck radiation, MRONJ at the extraction site, and poor image quality that would impair the image assessment were excluded.

Additionally, a control group was selected to match the study group regarding age, gender, and extracted tooth. Patients with a CBCT and tooth extractions within a year without a history of antiresorptive medication use were included in this group. Further exclusion criteria were the same as for the study group. Tooth extractions were performed following the description of Moreno-Rabié et al.^16^

### Data selection

Medical records (*i.e.* clinical data and CBCT images) were revised. The following information was retrieved: year of birth, gender, systemic condition, concomitant medication, previous chemotherapy and/or radiotherapy (*i.e.* other than to the head and neck region), ARD, dose, treatment duration, smoking status,^20^ alcohol habits, date of CBCT acquisition, extracted teeth, indication for extraction, surgery date, duration of follow-up, and if applicable, the date of diagnosis and stage of MRONJ according to the American Association of Oral and Maxillofacial Surgeons,^7^ site of MRONJ, drug holiday (*i.e.* treatment interruption before the tooth extraction), use of leukocyte- and platelet-rich fibrin (L-PRF), prophylactic antibiotics, antiseptic mouthwash, and date when mucosal healing was reached.

### Radiographic assessment

CBCT images were acquired with 3D Accuitomo 170 (J. Morita Corp., Kyoto, Japan) or Newtom VGi evo (Cefla Dental Group, Imola, Italy). The field of view (FOV), voxel size (ranging from 80 to 300µm), and exposure protocol for each exam were determined according to the patient’s specific diagnostic or therapeutic indication. All images were assessed using IMPAX software (v. 6.5.5, Agfa-Gevaert, Mortsel, Belgium).

Three blinded and independent oral and maxillofacial radiologists evaluated all images and scored the parameters explained below at each tooth extraction site. A calibration session took place before the start of the observations, using a set of 21 CBCTs external to the study to achieve baseline diagnostic consensus. All evaluations were done in a quiet room with low lighting using a high-resolution display (HP EliteDisplay E243 23.8-inch Monitor; HP Inc.; Palo Alto). The brightness and contrast settings were adapted according to the examiner’s judgment. 48 tooth extraction sites were reassessed 1 month after the evaluation was completed to determine the intraobserver agreement. The outcome was calculated using the mode of the observations, which meant that at least two of the observers had to agree on whether a characteristic was present or absent. Individual sites with no concordance were discussed until agreement was achieved.

**Figure 1.**
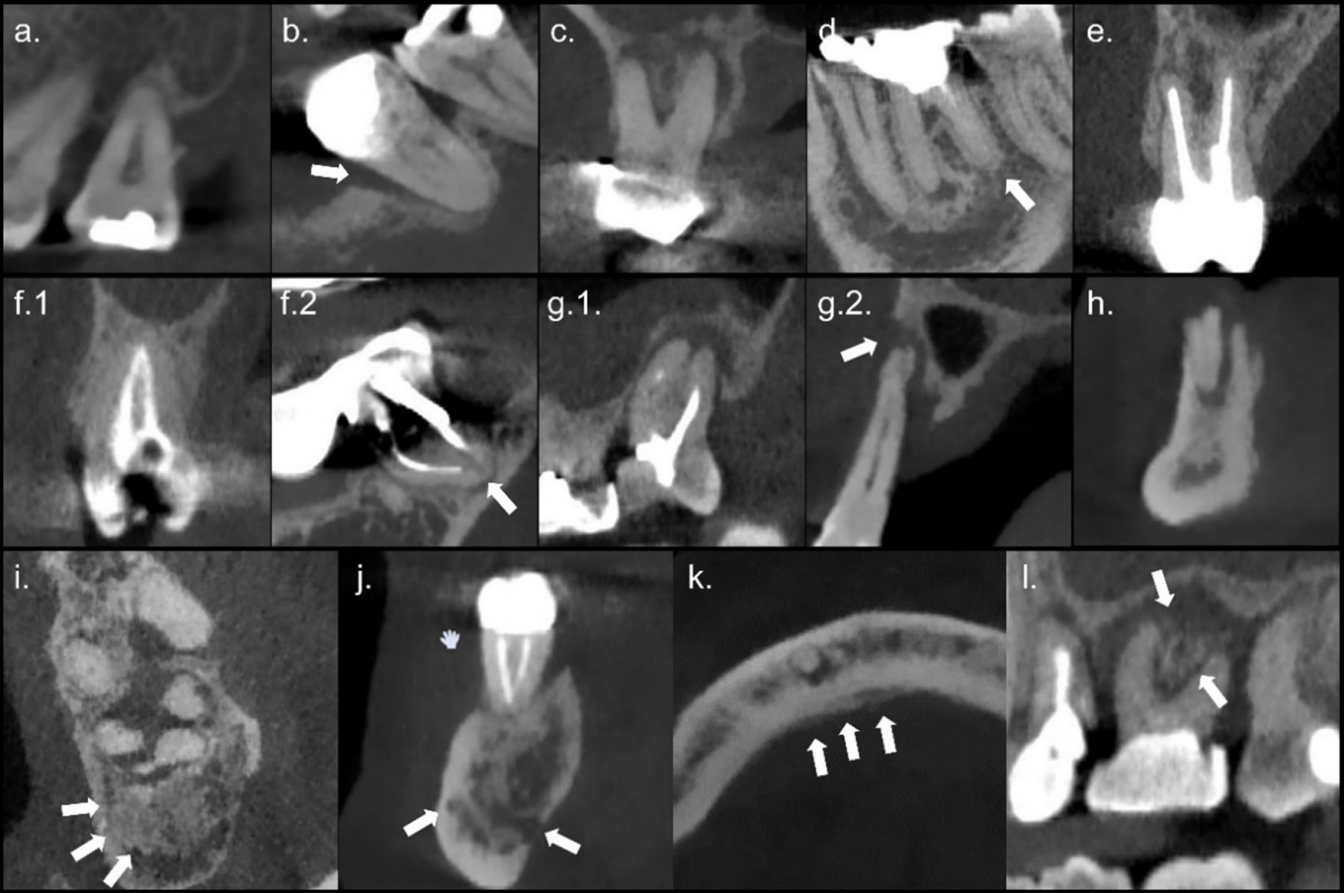
Cut-offs of CBCT reconstructions showing the assessed parameters. These images illustrate severe horizontal bone loss (**a**), an angular bone defect (**b**), furcation involvement (**c**), thickening of the lamina dura (**d**), widening of the periodontal ligament space (**e**), an adequate (f.1) and inadequate (f.2) endodontic treatment, a large periapical lesion with cortical expansion (g.1) and with buccal cortical plate destruction (g.2), a root remnant (**h**), osteosclerosis (**i**), osteolysis (**j**), periosteal reaction (**k**), and sequestrum formation (**l**).

Radiological evaluation included an examination of the tooth to be extracted and the surrounding bone (medullary and cortical bone), excluding the crown due to artifacts generated by high-density materials (*e.g.* fillings and metallic crowns) that prevented its proper visualization. The parameters assessed are shown in Figure 1 and listed hereafter:

1. Alveolar bone loss, considering the absence or presence of horizontal bone loss and angular bone defects as described by Gaeta-Araujo et al.^18^ In multirooted teeth, it was considered the worst outcome.
2. Furcation involvement, classified as not applicable/absent or present.
3. Lamina dura, normal or thickened.
4. Periodontal ligament space, normal or widened, if doubled in width.
5. Endodontic treatment, following the description of Nascimento et al,^21^ was described as absent, present with adequate filling, or present with inadequate filling, if underfilling of more than 2 mm coronal to the apex, overfilling, non-homogeneous filling, non-filled canal, presence of fractured instruments in the canal, or deviation of the natural course of the canal.
6. Periapical lesion, considering presence, size, and involvement of the cortical bone, based on the description of Fontenele et al.^22^ A lesion was deemed present if there was hypodensity in the periapical area wider than 1 mm. They were classified as small if their largest diameter was ≤3 mm or large if >3 mm. Cortical involvement was divided into four categories: none, thinning, expansion, and destruction.
7. Root remnant, absent or present.
8. Trabecular bone pattern, considering osteosclerosis, osteolysis, periosteal reaction, and sequestrum formation, based on the description of Walton et al.^23^ All characteristics were classified as absent, localized if only in the examined tooth, or extensive if involving further than the immediate neighboring tooth.

**Figure 2.**
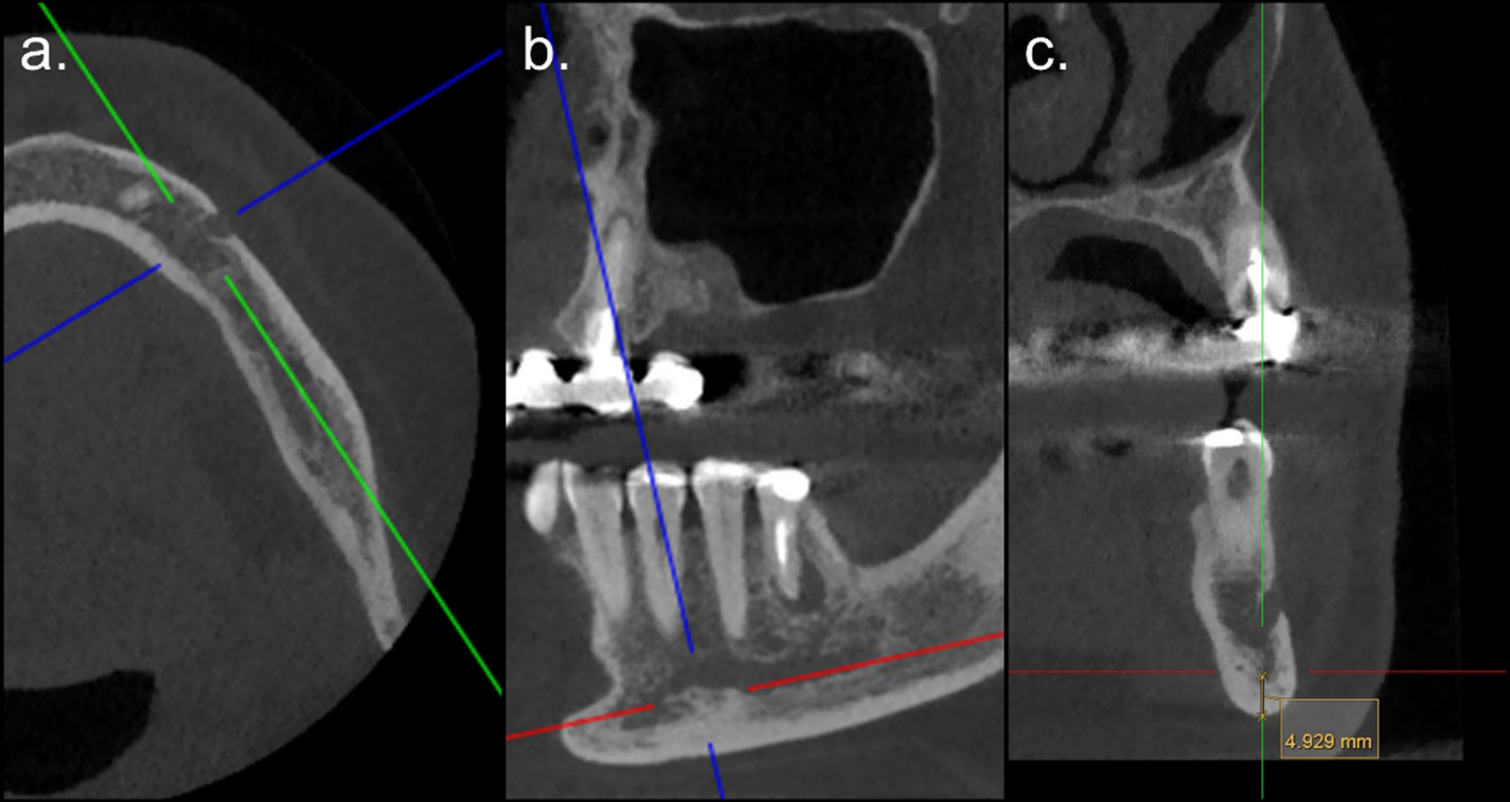
Example of measurement of the mandibular cortical width from the left mandibular side. The measurement was performed after completing three steps. (**a**) First, the axial reconstruction was selected where the largest dimension of the mental foramen was visible. Then, the orientation lines were rotated so that they could pass through the long axis of the mandible’s body and its tangent through the middle of the foramen. (**b**) Once these lines were in place, the line of the sagittal reconstruction was modified so that it would be parallel to the base of the mandible. (**c**) Finally, the MCW was measured in the coronal reconstruction using a line that passed through the posterior border of the foramen.

Furthermore, measurements of the mandibular cortical width (MCW) were performed once per side per patient to compare control and study groups, and within the latter, MRONJ+ and MRONJ- patients. Figure 2 depicts the measurement methodology adopted based on the description of Castro et al.^24^

### Statistical analysis

The statistical analysis was performed using the software RStudio v. 2023.3.1.446 (RStudio, Boston, MA), and a *p*-value ≤ 0.05 was considered significant. Fleiss’ κ test was used to calculate interobserver agreement, and Cohen’s κ test for intraobserver agreement. The results of the κ tests were interpreted according to the following scale: ≥0.21–0.40 was considered fair; moderate when it was ≥0.41–0.60; substantial when it was ≥0.61–0.80; almost perfect when it was ≥0.81–0.99.^25^ Furthermore, the χ^2^/Fisher’s exact test, for categorical data, and the Mann–Whitney *U* test, for ordinal variables, were used to test the independence of radiographic characteristics and clinical data documented for each patient/extracted tooth. In these analyses, control and antiresorptive-treated groups were compared. Additionally, comparisons were made by splitting the study group into extraction sites MRONJ+ and MRONJ-. Finally, the radiographic characteristics associated with the use of bisphosphonates and denosumab were investigated. For this purpose, patients who had only been exposed to one type of drug were selected, and the aforementioned tests were used to compare the distribution of radiographic features.

## Results

Forty-seven oncologic patients who had 87 teeth extracted met the inclusion criteria. MRONJ was observed in 15 patients (32%) and involved 33 sites (38%). In addition, the control group included 50 patients who had 106 tooth extractions, which showed no significant differences in age (*p* = 0.218), gender (*p* = 0.941), number of teeth to be extracted (*p* = 0.480), tooth type (*p* = 0.643), and arch (*p* = 0.201) with the study group. Other post-extraction complications included one bleeding and three oroantral communications, all successfully treated. Table 1 shows a summary of the patient’s data. The time between CBCT acquisition and tooth extraction ranged from 0 to 12 months, with an average time of 2 months for oncologic patients and 1 month for control patients.

**Table 1.** Summary of data collected at a patient level for oncologic and control groups

Characteristic		Oncologic						Control	
**Number of patients, n**		47						50	
**Development of osteonecrosis, n (%**)		MRONJ+		MRONJ-		Total	*p*-value	NA	*p*-value
		15	31.9%	32	68.1%	47			
**Age (years**)	Mean (range)*	63.8 (46– 83)		71.3 (46– 89)		68.9 (46–89)	*0.025*	71.5 (47–87)	0.218
**Sex, n (%**)	Female	10	40%	15	60%	25	0.340	28	0.941
	Male	5	22.7%	17	77.3%	22		22	
**Extracted teeth, n**	Mean (range)*	2.8 (1–8)		1.4 (1–4)		1.9 (1– 8)	*0.006*	2.1 (1–6)	0.480
**Chemo- and radiotherapy, n (%)^a^**	None	1	50%	1	50%	2	0.603	43	*<0.001*
	Chemotherapy	4	44.4%	5	55.6%	9		2	
	Radiotherapy	2	20%	8	80%	10		1	
	Both	8	30.8%	18	69.2%	26		4	
**ARD type, n (%**)	Bisphosphonate	6	28.6%	15	71.4%	21	0.779	NA	NA
	Denosumab	8	33.3%	16	66.7%	24		NA	
	Both	1	50%	1	50%	2		NA	
**Specific ARD used, n (%**)	Zoledronic Acid	5	23.8%	16	76.2%	21	0.467	NA	NA
	Denosumab	9	34.6%	17	65.4%	26		NA	
	Alendronate	1	100%	0	0%	1		NA	
	Pamidronate	1	33.3%	2	66.7%	3		NA	
	Ibandronate	1	50%	1	50%	2		NA	
Number of ARD, n (%)*	1	13	31.7%	28	68.3%	41	0.953	NA	NA
	2	2	33.3%	4	66.7%	6		NA	
**Time on ARD (months**)	Mean (range)*	40.4 (7– 173)		29.5 (1– 86)		32.9 (1–173)	0.568	NA	NA
**Drug holiday (months), n (%**)	No	5	38.5%	8	61.5%	13	0.728	NA	NA
	Yes	10	29.4%	24	70.6%	34		NA	
	Mean (range)*	7.8 (0.1–29)		27.5 (0.3–119)		21.6 (0.1–119)	*0.021*	NA	
**Corticosteroid use (months), n (%**)	No	8	26.7%	22	73.3%	30	0.484	48	*<0.001*
	Yes	7	41.2%	10	58.8%	17		1	
	Mean (range)*	38.5 (10– 96)		61.3 (3– 420)		51.9 (3–420)	0.115	2.5	0.191
**Osteoporosis, n (%**)	No	11	30,6%	25	69,4%	36	0.725	46	0.069
	Yes	4	36,4%	7	63,6%	11		4	
Alcohol consumption, n (%)*	No consumption	6	40%	9	60%	15	0.293	16	0.365
	1–2 units week	1	10%	9	90%	10		17	
	3–4 units week	0	0%	0	0%	0		4	
	>5 units week	3	42%	4	57.1%	7		8	
	Ex-abuser	0	0%	2	100%	2		1	
	Unknown	5	38.5%	8	61.5%	13		4	
**Tobacco use, n (%)^c^**	Never smoker	5	22.7%	17	77.3%	22	*0.004*	36	*0.034*
	Current smoker	5	100%	0	0%	5		7	
	Former smoker	4	25%	12	75%	16		7	
	Unknown	1	25%	3	75%	4		0	
MCW*	Right	4.11		4.72		4.50	0.071	4.23	0.247
	Left	4.32		4.70		4.56	0.190	4.15	0.071

ARD: antiresorptive drug,CDC, Center for Disease Control and Prevention; MCW, mandibular cortical width; MRONJ: medication-related osteonecrosis of the jaws, NA: not applicable.

*p*-values represent the results of the χ^2^/Fisher’s exact test when comparing MRONJ+ and MRONJ- patients in the study group, as well as the study and control groups.

Variables denoted with an asterisk (*) represent ordinal/numerical data analyzed with the Mann–Whitney *U* test.

Significant *p*-values (*p* ≤ 0.05) are *italicized*.

a No head and neck radiotherapy or ARDs were given to control patients with a history of cancer.

b Referring to the number of different ARDs used sequentially.

c Following the definition provided by the CDC from the United States, which states as never smoker a person who has never smoked or has smoked less than a 100 cigarettes in their lifetime, as current smoker a person who has smoked at least 100 cigarettes in their lifetime and currently smokes, and as former smoker a person who has smoked at least a 100 cigarettes in their lifetime but who had quit at the time of interview.

Overall, observers had a substantial agreement between their assessments (*k* = 0.69), ranging from a moderate agreement in periodontal ligament space and osteolysis (*k* = 0.41) to almost perfect agreement in endodontic treatment (*k* = 0.95). Furthermore, no significant differences were found among the observer’s measurements of mandibular cortical width (right side *p* = 0.87; left side *p* = 0.96). Finally, the intraobserver agreement had an almost perfect concordance (*k* = 0.87).

Patients with malignant disease received at least one dose of zoledronic acid 4 mg, denosumab 120 mg, or pamidronate 90 mg. These patients were diagnosed with breast cancer (*n* = 19, 40.4%), multiple myeloma (*n* = 11, 23.4%), prostate (*n* = 10, 21.3%), renal cell (*n* = 3, 6.4%), lung (*n* = 2, 4.3%), stomach (*n* = 1, 2.1%), and pancreatic cancer (*n* = 1, 2.1%). There were no significant differences in the type of cancer and the onset of osteonecrosis of the jaws (*p* = 0.642) nor in the type of ARD used (*p* = 0.779) or the length of treatment (*p* = 0.568) (Table 1). Additionally, complementary cancer therapies at the time of tooth extraction such as the use of anti-angiogenic drugs (*n* = 9, *p* = 1.000) and hormone therapy (*n* = 17, *p* = 0.961) also failed to demonstrate a significant effect on the development of MRONJ.

Patients who developed MRONJ had a significantly shorter drug holiday than those without exposed bone (*p* = 0.021). Furthermore, when bisphosphonates and denosumab were studied separately, patients on bisphosphonates had a mean drug holiday of 36 months (MRONJ+: 14 months and MRONJ-: 44 months; *p* = 0.094), while those on denosumab had a mean drug holiday of 8 months (MRONJ+: 1.9 months and MRONJ-: 11 months; *p* = 0.041). Moreover, a younger age (MRONJ+ mean 64 years, MRONJ- mean 71 years, *p* = 0.025), multiple tooth extractions simultaneously (MRONJ+ mean three teeth, MRONJ- mean one tooth, *p* = 0.006), and smoking (*p* = 0.004) significantly increased the risk of developing MRONJ.

The clinical variables studied for each extracted tooth are detailed in Table 2, and the results of the radiographic assessment are displayed in Table 3. None of the clinical factors were found to be significant in the onset of osteonecrosis. It was noted that mucosal lining at the extraction site was achieved on an average of 2.4 weeks in the control group. In contrast, the study group took significantly longer for this sign to develop, averaging 14.2 weeks post-extraction (*p* < 0.001).

**Table 2.** Description of patient data collected per extracted tooth in the oncologic and study groups

Characteristic		Oncologic						Control	
**Number of extracted teeth, n**		87						106	
**Development of osteonecrosis, n (%**)		MRONJ+		MRONJ-		Total	*p*-value	NA	*p*-value
		33	37.9%	54	62.1%	87			
**Extraction indication, n (%**)	Caries	20	48.8%	21	51.2%	41	0.338	45	*<0.001*
	Periodontitis	11	34.4%	21	65.6%	32		16	
	Fracture	0	0%	2	100%	2		11	
	Root remnant	2	22.2%	7	77.8%	9		25	
	Pericoronitis	0	0%	1	100%	1		7	
	NA	0	0%	2	100%	2		2	
Type of teeth, n (%) *	Incisors + canines	6	28.6%	15	71.4%	21	0.843	30	0.643
	Premolars	11	57.9%	8	42.1%	19		21	
	Molars	16	34%	31	66%	47		55	
**Arch, n (%**)	Maxilla	14	29.8%	33	70.2%	47	0.140	68	0.201
	Mandible	19	47.5%	21	52.5%	40		38	
**Region, n (%**)	Anterior maxilla	3	27.3%	8	72.7%	11	0.221	18	0.459
	Posterior maxilla	11	30.6%	25	69.4%	36		50	
	Anterior mandible	3	30%	7	70%	10		12	
	Posterior mandible	16	53.3%	14	46.7%	30		26	
**Antibiotic prophylaxis, n (%**)	Yes	30	36.1%	53	63.9%	83	0.151	20	*<0.001*
	No	3	75%	1	25%	4		86	
**Antiseptic mouthwash, n (%**)	Yes	33	37.9%	54	62.1%	87	1.000	106	1.000
	No	0	0%	0	0%	0		0	
**Use of L-PRF, n (%**)	Yes	24	33.8%	47	66.2%	71	0.166	4	*<0.001*
	No	9	56.3%	7	43.8%	16		102	
**Time until mucosal healing, n (%**)	0–≤4 weeks	0	0%	26	100%	26	NA	99	*<0.001*
	>4–≤8 weeks	0	0%	28	100%	28		7	
	>8 weeks	33	100%	0	0%	33		0	
	Mean (weeks)*	42.8		4.09		14.19	*<0.001*	2.41	*<0.001*
**MRONJ worse stage, n (%**)	Stage 1	21	63.6%	NA		NA	NA	NA	NA
	Stage 2	10	30.3%	NA		NA		NA	
	Stage 3	2	6.1%	NA		NA		NA	

L-PRF, leukocyte- and platelet-rich fibrin; MRONJ, medication-related osteonecrosis of the jaws; NA, not applicable.

The *p*-values described under oncologic and control correspond to those obtained with the χ^2^/Fisher’s exact test or Mann–Whitney *U* test when data were ordinal (*).

Comparisons were made between MRONJ+ and MRONJ-sites, and study and control groups.

Significant *p*-values (*p* ≤ 0.05) are *italicized*.

**Table 3.** Results of the pre-operative CBCT assessment at each tooth extraction site in the study and control groups

Observed parameter		Oncologic						Control	
**Number of extracted teeth, n**		87						106	
**Development of osteonecrosis, n (%**)		MRONJ+		MRONJ-		Total	*p*-value	NA	*p*-value
		33	37.9%	54	62.1%	87			
**Horizontal bone loss**	Absent/initial	21	35.6%	38	64.4%	59	0.678	81	0.242
	Moderate/severe	12	42.9%	16	57.1%	28		25	
**Angular bone defect**	Absent	26	37.1%	44	62.9%	70	0.977	83	0.850
	Present	7	41.2%	10	58.8%	17		23	
**Furcation involvement**	Absent	27	39.7%	41	60.3%	68	0.705	86	0.740
	Present	6	31.6%	13	68.4%	19		20	
**Lamina dura**	Normal	21	36.2%	37	63.8%	58	0.815	86	*0.033*
	Thickened	12	41.4%	17	58.6%	29		20	
**Periodontal ligament space**	Normal	8	42.1%	11	57.9%	19	0.875	51	*<0.001*
	Widened	25	36.8%	43	63.2%	68		55	
**Endodontic treatment**	Absent	20	36.4%	35	63.6%	55	0.905	65	0.955
	Adequate filling	5	38.5%	8	61.5%	13		16	
	Inadequate filling	8	42.1%	11	57.9%	19		25	
Periapical lesion size*	Absent	16	31.4%	35	68.6%	51	0.229	66	0.593
	Small (≤3 mm)	7	58.3%	5	41.7%	12		14	
	Large (>3 mm)	10	41.7%	14	58.3%	24		26	
Periapical lesion cortical*	Absent	16	31.4%	35	68.6%	51	0.193	66	0.607
	None	5	50%	5	50%	10		12	
	Thinning	4	44.4%	5	55.6%	9		7	
	Expansion	3	75%	1	25%	4		8	
	Destruction	5	38.5%	8	61.5%	13		13	
**Root remnant**	Absent	32	39.5%	49	60.5%	81	0.401	85	*0.018*
	Present	1	16.7%	5	83.3%	6		21	
Osteoclerosis*	Normal	8	32%	17	68%	25	0.247	51	*0.006*
	Localized sclerosis	2	22.2%	7	77.8%	9		10	
	Extended sclerosis	23	43.4%	30	56.6%	53		45	
Osteolysis*	Absent	26	36.6%	45	63.4%	71	0.546	102	*<0.001*
	Localized lysis	4	36.4%	7	63.6%	11		3	
	Extensive lysis	3	60%	2	40%	5		1	
Periosteal reaction*	Absent	29	35.4%	53	64.6%	82	0.051	104	0.155
	Localized reaction	2	100%	0	0%	2		1	
	Extensive reaction	2	66.7%	1	33.3%	3		1	
Sequestrum formation*	Normal	32	38.1%	52	61.9%	84	0.879	106	0.055
	Localized sequester	1	33.3%	2	66.7%	3		0	
	Extensive sequester	0	0%	0	0%	0		0	

NA: not applicable.

*p*-values represent the results of the χ^2^/Fisher’s exact test when comparing MRONJ+ and MRONJ- patients in the study group, as well as the study and control groups.

Variables denoted with an asterisk (*) represent ordinal/numerical data analyzed with the Mann–Whitney *U* test.

Significant *p*-values (*p* ≤ 0.05) are *italicized*.

Concerning the radiographic signs predisposing to MRONJ, the presence of localized and extensive periosteal reaction was associated with a higher risk of bone exposure compared to its absence (*p* = 0.051). All teeth in sites showing periosteal reaction, presented extensive caries lesions, periapical radiolucencies and/or periodontitis, accompanied by pain, increased response to cold stimuli, tenderness to percussion, periapical fistula, or abscess formation. Interestingly, sequester formation was exclusively seen in the study group, both in sites that later did and did not develop MRONJ. All sites with sequester formation were also accompanied by teeth with caries or periodontal disease, and presenting tenderness to percussion or mobility, respectively.

**Figure 3.**
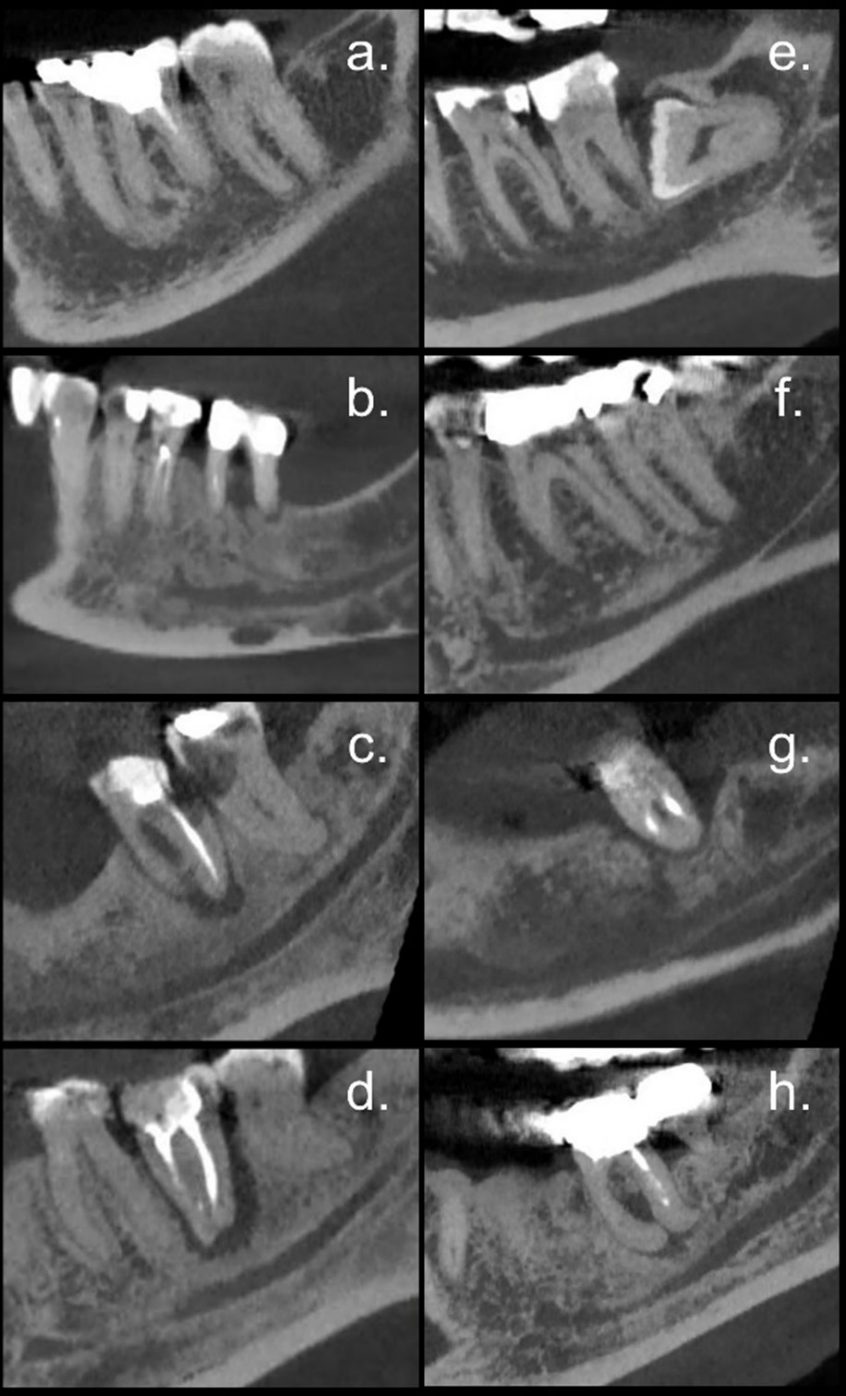
Sagittal reconstruction cuts showing trabecular and cortical bone in the mandibular molar areas of patients treated with bisphosphonates (**a–d**) and denosumab (**e–h**). Patients treated with bisphosphonates had significantly more localized (**a**) or extensive (**b, c, d**) osteosclerosis, whereas those treated with denosumab showed more frequently normal medullary spaces (**e, f**) and less often localized (**g**) or extensive (**h**) osteosclerosis.

Finally, among the oncologic patients, 21 received only bisphosphonates, while 24 received denosumab. There were 42 extractions in each group. Results showed no significant differences in the distribution of lamina dura appearance (*p* = 0.646), periodontal ligament space (*p* = 0.602), osteolysis (*p* = 0.401), periosteal reaction (*p* = 0.180), and sequestrum formation (*p* = 0.568) when comparing both types of medication. However, patients who received bisphosphonates (BP) had significantly more localized and extensive osteosclerosis than those who received denosumab (DB) (localized: 12% in BP *vs* 9% in DB, extensive: 74% in BP *vs* 45% in DB, *p* = 0.003) (Figure 3).

## Discussion

Currently, there is no widely agreed recommendation on the best imaging approach for detecting patients at increased risk of clinically overt MRONJ. As a result, osteonecrosis lesions are often only evaluated when clinically exposed bone is present, overlooking early radiographic alterations and potential risk factors.^8,11,26^ Studies have revealed that exposed bone locations may exhibit earlier symptoms of infection or trabecular bone alterations.^16–18^ However, all these studies were performed using panoramic radiographic assessment, whose inherent limitations include overlapping of anatomical structures, magnification, and absence of a buccolingual evaluation, which might impair their diagnostic performance. Thus, our primary goal was to identify clinical and local predisposing factors in oncologic patients treated with ARD and undergoing tooth extractions using three-dimensional images.

The use of CBCT for diagnosing MRONJ is advocated due to its better resolution than panoramic radiography, the possibility of assessing the true extent of a lesion, and the visibility of structures without overlap.^11^ Through CBCT assessment, it has been possible to observe the presence of osteolysis, cortical bone erosion, sequestrum formation, and osteosclerosis in lesions with bone exposure at all clinical stages as defined by the American Association of Oral and Maxillofacial Surgeons.^7^ Adding to these results, the present investigation describes the distinguishing three-dimensional features of ARD use even before the presence of clinically exposed necrotic bone. Given that our patients demonstrated significantly more thickening of the lamina dura, widening of the periodontal ligament space, osteosclerosis, osteolysis and sequestrum formation than the control group. All these findings are consistent with prior two-dimensional investigations.^9,17,27,28^

Regarding clinical risk factors associated with osteonecrosis exposure, a higher risk was seen in younger patients, with more than one simultaneous tooth extraction, smokers, and with shorter discontinuation of ARDs when the tooth extractions took place. The latter is particularly important in light of the pharmacokinetics of BP and DB, which have half-lives of up to 10 years^29^ and 1 month,^30^ respectively. Besides, BPs, unlike DB, are deposited in bone tissue and only exert their effect on osteoclasts at the time of their uptake.^29^ This explains why patients who had stopped taking BP for an average of 14 months and DB for an average of 2 months at the time of tooth extractions developed osteonecrosis.

Knowing that ARDs have distinct modes of action, it is conceivable that the radiographic characteristics of osteonecrosis related to bisphosphonate- (BRONJ) and denosumab- (DRONJ) are distinct.^31^ Pichardo et al found significant differences in their radiographic appearance, with BRONJ having significantly more sequester formation and cortical bone osteolysis and DRONJ showing less frequent radiographic signs leading to a later diagnosis and treatment.^32^ In our results, even before the development of MRONJ, sites exposed to bisphosphonates had significantly more osteosclerosis, while those exposed to denosumab showed no significant features. The latter is relevant because the absence of early radiographic differences in denosumab-treated patients could affect their timely follow-up, as they tend to show late signs.^32^

Periosteal reaction yielded a borderline significance, suggesting that its presence alone may not conclusively predict the development of an exposed variant of necrosis. Nonetheless, sites exhibiting periosteal reaction could potentially harbor latent osteonecrosis lesions. Although this study lacks histopathological evidence, the use of ARD, coupled with the subsequent impairment of bone’s reparative response, in combination with dental infections, creates a favorable environment for osteonecrosis.^33^ Thus, the possibility of a masked necrotic process cannot be ruled out. Additionally, even though not significant for MRONJ, bone sequestrum was observed only in the study group. Barragan-Adjemian et al described in CBCTs that bone islands surrounded by an osteolytic halo were a natural response to expel necrotic bone in the direction where there was the least resistance, resulting in clinical MRONJ.^13^ We hypothesize that having bone exposure at these sites was a matter of timing, and very likely, in the MRONJ- sites, surgical removal of sequesters during tooth extraction was curative.^34,35^

Variations in the surgical technique could affect the occurrence of osteonecrosis even when all patients are exposed to the same risk factor. According to Seidel et al, tooth extractions combined with alveolectomy and the use of platelet-rich fibrin membranes significantly decreased the incidence of MRONJ. It is believed that surgically removing the alveolar bone, which is anticipated to remodel, would aid healing and reduce the risk of osteonecrosis.^36^ Despite surgical variability, most patients included in the current investigation had dental extractions under antibiotic prophylaxis (amoxicillin 875 mg/clavulanic acid 125 mg or clindamycin 300 mg), local anesthetic without vasoconstrictor, L-PRF membranes, and resorbable sutures. All factors that could decrease the incidence of MRONJ. Yet, regardless of preventive measures, the study group took longer to achieve mucosal coverage than the control group. Under a normal setting, the mucosal lining of the exposed post-extraction socket is expected within 2 weeks,^37–40^ but it took a mean of 14 weeks in the ARD-treated patients. Similar evidence supporting BP-use to heal at a slower rate after tooth extractions had been reported with a median of 5 weeks.^40^

The retrospective design of our study has some limitations, such as missing data in patient files, surgical variability, different ARD treatments, polypharmacy, and comorbidity factors, among other variables that may impact the development of osteonecrosis. However, this design allowed us to include a larger number of patients meeting our criteria. Specifically, we studied 47 ARD-treated patients who had 85 tooth extractions, from which 33 sites developed MRONJ. The high incidence of MRONJ can be attributed to two factors. Firstly, University Hospitals Leuven is a specialized referral center for ARD-treated patients, resulting in a higher concentration of MRONJ cases. Secondly, all included patients were exposed to a well-known risk factor for MRONJ, which is tooth extraction. Furthermore, the radiographic assessment was performed on a localized area, meaning that osteonecrosis lesions on the opponent quadrant from the assessed teeth, which may have been the reason for CBCT acquisition in the first place, did not impair the assessment of local risk factors.

Periosteal reaction and sequestrum formation are imaging features which may be indicative of osteonecrosis by CBCT assessment. Further studies with larger samples are required to explore these local radiographic features and their histopathological correlation. Nevertheless, by demonstrating significant variations between the study and control groups, ARD administration was found to cause trabecular bone alterations. Interestingly, individuals treated with bisphosphonates and denosumab exhibited differential trabecular bone patterns. When it comes to clinical advice based on the present sample for the prevention of osteonecrosis in oncological patients, we urge first and foremost prevention, the abstention of smoking, and periodic dental examinations to avoid multiple extractions. For tooth extractions during ARD treatment, consider the drug pharmacokinetics before discontinuation, as a brief pause will not reduce the risk and the presence of infection may even increase the likelihood of osteonecrosis. Lastly, treatment continuation can outweigh the risk for MRONJ due to the potential for fractures and metastatic progression.^41^

## Conclusion

The findings suggest that periosteal reaction on CBCT may indicate an elevated risk or possibly a latent MRONJ in oncologic patients. Similarly, sequestrum formation was exclusively seen in the ARD-treated patients and is also suspected of being a pre-clinical indicator of MRONJ. Additionally, the use of ARDs can lead to bony changes, and the type of ARD used may influence the radiographic variations observed.
